# Genetic Diversity of *Cryptosporidium parvum* in Neonatal Dairy Calves in Xinjiang, China

**DOI:** 10.3390/pathogens9090692

**Published:** 2020-08-23

**Authors:** Yayun Wu, Kuankuan Zhang, Ying Zhang, Bo Jing, Yuancai Chen, Chunyan Xu, Tian Wang, Meng Qi, Longxian Zhang

**Affiliations:** 1 College of Animal Science, Tarim University, Alar 843300, China; yayunwu@cau.edu.cn (Y.W.); ca6856371@gmail.com (K.Z.); yingzhang782@gmail.com (Y.Z.); 120050010@taru.edu.cn (B.J.); 120170015@taru.edu.cn (C.X.); 120180029@taru.edu.cn (T.W.); 2 College of Animal Science and Veterinary Medicine, Henan Agricultural University, Zhengzhou 450046, China; chenyc217@gmail.com

**Keywords:** *Cryptosporidium*, neonatal dairy calves, diarrhea, subtype, Xinjiang

## Abstract

*Cryptosporidium parvum* has been identified as a major cause of diarrhea and diarrhea-associated deaths in young children and neonatal calves. Infections can remain asymptomatic but may lead to malnutrition and persistent growth retardation. To assess the relationship between *C. parvum* genetic diversity and pathogenicity in neonatal dairy calves and determine the cause of diarrhea among these calves, 232 fecal samples from neonatal dairy calves on 12 farms in Xinjiang, China, were characterized for *Cryptosporidium* presence based on the small subunit rRNA gene. The *Cryptosporidium* prevalence was 38.4% (89/232), and three species were detected with restriction fragment length polymorphism analysis, including *C. parvum* (the significantly dominant species), *C. ryanae*, and *C. bovis*. *Cryptosporidium* prevalence was significantly higher in neonatal dairy calves with diarrhea (52.6%, 51/97) than in calves without diarrhea (28.1%, 38/135). All *C. parvum*-positive samples were analyzed based on the 60 KDa glycoprotein gene, and IIdA15G1, IIdA20G1, IIdA14G1, and IIdA19G1 were successfully subtyped. These data indicate that *C. parvum* may be a major contributor to diarrheal disease in neonatal dairy calves, and *C. parvum* subtypes from neonatal dairy calves in Xinjiang exhibited high genetic diversity.

## 1. Introduction

*Cryptosporidium* spp. are common causative pathogens of gastroenteritis in humans and animals and is second only to the rotavirus as the causative pathogens of moderate-to-severe diarrhea in children aged younger than 2 years in the developing world [1,2]. Currently, at least 42 valid *Cryptosporidium* species have been recognized, and *C. homisis* and *C. parvum* cause most of the infections in humans [3,4]. Many studies have focused on cattle, with preweaned dairy calves being considered the most important reservoir for zoonotic infection [1,5]. Among common infections with *C. parvum*, *C. bovis*, *C. andersoni*, and *C. ryanae* in cattle, *C. parvum* has been associated with clinical disease in neonatal dairy calves, and *C. parvum* pathogenicity may be a consequence of host–microbe interactions from a long-term evolutionary perspective [6,7]. Moreover, *C. xiaoi, C. meleagridis, C. hominis,* and *C. tyzzeri* have been identified in dairy cattle in China. The overall infection rate of *Cryptosporidium* spp. in dairy cattle in China is 14.0%, with *C. parvum* and *C. bovis* being the predominant species [5].

Nearly 20 *C. parvum* subtype families have been identified via phylogenetic analysis of the 60-KDa glycoprotein (*gp60*, also known as *gp40/15*) gene [4,8]. Compared with other countries, accumulating evidence shows a uniqueness of *C. parvum* subtype distributions in preweaned dairy calves in China. Early work has pointed to *C. parvum* IId subtypes being predominant in dairy calves in China, whereas *C. parvum* IIa subtypes were predominant in preweaned dairy calves in Europe, North America, and Australia [5,8]. One study demonstrated that *C. parvum* IId subtypes likely dispersed from Western Asia to other geographical regions through cattle introduction [9]. At least six *C. parvum* IId subtypes (IIdA14G1, IIdA15G1, IIdA17G1, IIdA19G1, IIdA20G1, and IIdA20G1) have been detected in dairy cattle in China [5]. Several studies have revealed that *C. parvum* subtype IIdA19G1 is the most commonly detected subtype in dairy cattle in eastern and central China. Additionally, cryptosporidiosis outbreaks were reported on two dairy farms in the Ningxia Hui Autonomous Region (hereinafter referred to as Ningxia) and Jiangsu Province, which were caused by *C. parvum* subtypes IIdA15G1 and IIdA19G1, respectively [7,10].

Located in northwest China (73°40′ E–96°18′ E, 34°25′ N–48°10′ N), Xinjiang Uygur Autonomous Region (hereinafter referred to as Xinjiang) is the largest Chinese administrative division, spanning over 1.6 million km^2^ (0.64 million square miles). Historically, Xinjiang was a necessary part of the cattle trading route between Central Asia and China, and it presented a unique geographical advantage in the processes of cattle domestication and breeding. Holstein dairy cattle have been intensively farmed on a large scale in Xinjiang for at least 30 years. Unlike other provinces and municipalities in China, *C. parvum* subtypes IIdA14G1 and IIdA15G1 have been detected in dairy calves in Xinjiang [11]. To further address the knowledge gap regarding *C. parvum* genetic diversity and pathogenicity in this region, this study was conducted to examine the occurrence of *Cryptosporidium* spp. and determine the relationship between *C. parvum* subtypes and diarrhea in neonatal dairy calves and to assess the zoonotic transmission risk of *C. parvum*.

## 2. Results

### 2.1. Prevalence of Cryptosporidium spp. in Neonatal Dairy Calves

*Cryptosporidium* spp. were detected in 89 of 232 fecal samples (38.4%) as per *SSU* rRNA gene sequencing. *Cryptosporidium* spp. were detected on all 12 farms, with the highest prevalence in Tiemenguan (66.7%, 10/15) and the lowest prevalence in Hutubi (25.0%, 5/20; Table 1). *Cryptosporidium* prevalence did not significantly differ among farms (*p* > 0.05) but was significantly higher in neonatal dairy calves with diarrhea (52.6%, 51/97) than those without diarrhea (28.1%, 38/135; *p* < 0.05; Table 2).

Restriction fragment length polymorphism (RFLP) analysis revealed three *Cryptosporidium* species: *C. parvum* (*n* = 88), *C. ryanae* (*n* = 9), and *C. bovis* (*n* = 1; Table 1). *C. parvum* was the dominant species detected on all 12 farms. Nine *C. ryanae* isolates were detected in Shihezi and Tiemenguan. Only one *C. bovis* isolate was detected in Tiemenguan. Two *Cryptosporidium* species were detected in diarrheal neonatal dairy calves: *C. parvum* (*n* = 51) and *C. ryanae* (*n* = 1). Three *Cryptosporidium* species were detected in neonatal dairy calves without diarrhea: *C. parvum* (*n* = 37), *C. ryanae* (*n* = 8), and *C. bovis* (*n* = 1; Table 2).

Nine samples from three farms showed mixed infections. Coinfections of *C. parvum* and *C. ryanae* were identified in neonatal dairy calves with diarrhea (*n* = 1) and without diarrhea (*n* = 7); coinfections of *C. bovis* and *C. ryanae* were found only in neonatal dairy calves without diarrhea (Table 1; Table 2).

### 2.2. Distribution of C. parvum Subtypes

From 88 *C. parvum*-positive samples selected for subtyping, 86 (97.8%, 86/88) were successfully sequenced from the *gp60* gene. Four subtypes were identified; IIdA15G1 was the dominant subtype (40.7%, 35/86), followed by IIdA20G1 (24.4%, 21/86), IIdA14G1 (19.8%, 17/86), and IIdA19G1 (15.1%, 13/86; Table 1). The predominant subtype, IIdA15G1, was detected from seven farms. IIdA20G1 was identified from three farms, IIdA14G1 was detected on four farms, and IIdA19G1 was detected on two farms from Hutubi (Table 1).

IIdA15G1 was the dominant subtype in neonatal dairy calves with diarrhea (49.0%, 24/49), followed by IIdA20G1 (28.6%, 14/49), IIdA14G1 (14.3%, 7/49), and IIdA19G1 (8.2%, 4/49; *p* < 0.01; Table 2). IIdA15G1 was also the dominant subtype in neonatal dairy calves without diarrhea (29.7%, 11/37), followed by IIdA14G1 (27.0%, 10/37), IIdA19G1 (24.3%, 9/37), and IIdA20G1 (18.9%, 7/37; *p* > 0.05; Table 2).

## 3. Discussion

*Cryptosporidium* prevalence in preweaned calves is 3.4–96.6% worldwide [6]. In this study, *Cryptosporidium* spp. were detected on all 12 dairy cattle farms, and the overall prevalence was 38.4% (89/232). Compared with previous studies conducted in preweaned dairy calves aged < 2 months in China, the *Cryptosporidium* prevalence was similar to that in several reports from Shanghai Municipality (37.0%, 303/818) [12], Heilongjiang Province (33.2%, 86/259) [13], and Ningxia (31.0%, 49/158) [7]. The prevalence was higher than that reported for Shaanxi Province (24.7%, 46/186) [14], Guangdong Province (24.0%, 93/388) [15], Henan Province (21.5%, 172/801) [16], Hubei Province (15.8%, 42/265) [17], Xinjiang (15.6%, 37/237) [11], Sichuan Province (14.4%, 40/278) [18], Ningxia (14.0%, 122/871 and 10.2%, 19/186) [19,20], Guangdong Province (6.4%, 19/297) [21], Hebei Province, and Tianjin Municipality (2.6%, 9/351) [22]. Finally, the prevalence was lower than that in one case from Heilongjiang Province (47.7%, 72/151) [23].

Several reports have indicated that *C. bovis* is the predominant species in preweaned dairy calves in China, including reports from Shanghai Municipality [12], Guangdong Province [15,21], Henan Province [16], Shaanxi Province [14], Hubei Province [17], and Sichuan Province (<1 month) [18]. However, accumulating evidence suggests that *C. parvum* is the dominant species in preweaned dairy calves from Ningxia [7,19], Xinjiang [11], Beijing Municipality (<1 year) [24], Hebei Province, Tianjin Municipality [22], and Heilongjiang Province (<3 months) [13]. *C. parvum* mainly infects dairy calves within 1 month of age, while *C. bovis* and *C. ryanae* have been more commonly detected in 2–3-month-old dairy calves [6,13]. Species occurrence and distribution can be attributed to cattle age, specimen size, management systems, seasons, and geographic area. In this study, *C. parvum* was the dominant species, whereas *C. bovis* was only detected in one sample. In contrast, *C. parvum* is less prevalent than *C. bovis* in preweaned dairy calves in most studies conducted in China.

Four species of *Cryptosporidium* are commonly found in cattle: *C. parvum*, *C. bovis*, *C. ryanae* and *C. andersoni*, and more than 90% of the infection cases in preweaned dairy calves are attributed to *C. parvum*, which is reported to be a major cause of calf enteritis [4]. In China, severe diarrhea was observed in preweaned calves on a dairy farm in Ningxia in 2013, and *C. parvum* was the major cause of the outbreak [7]. Severe diarrhea was also reported in neonatal dairy calves on a large dairy farm in Jiangsu Province in 2016, and approximately 360 dairy calves died from watery diarrhea despite antibiotic therapy [10]. Additionally, in a longitudinal study in the USA, a group of dairy calves (*n* = 30), from birth to 24 months, exhibited the highest *Cryptosporidium* infection prevalence at 2 weeks of age, and *C. parvum* constituted 97% of the infections in the preweaned calves [25]. The *Cryptosporidium* (mainly *C. parvum*) infection rate was significantly higher in neonatal dairy calves with diarrhea than in calves without diarrhea in this study, further suggesting that *C. parvum* is associated with clinical disease in neonatal dairy calves. Generally, *C*. *bovis* and *C. ryanae* usually infect postweaned calves and yearlings, with no associated clinical disease [5,6]; however, the *C. ryanae* infection rate in preweaned dairy calves with diarrhea was significantly higher than in those without diarrhea on a farm that had a cryptosporidiosis outbreak in Jiangsu Province [10]. Therefore, more investigations are needed to clarify the *C. ryanae* pathogenicity for calves.

To date, seven *C. parvum* IId subtypes have been detected from cattle in China (Table 3), with subtypes IIdA14G1, IIdA15G1, IIdA19G1 and IIdA20G1 being zoonotic. IIdA15G1 was mostly found in Ningxia, Xinjiang, Heilongjiang Province, Sichuan Province, Beijing Municipality, and Gansu Province [7,11,13,18,19,20,24]. Subtype IIdA19G1 was mostly found in Jiangsu Province, Henan Province, Shanghai Municipality, Xinjiang, Guangdong Province, Hebei Province, Tianjin Municipality, Beijing Municipality, and Heilongjiang Province [10,12,15,16,22,23,24]. Subtype IIdA20G1 was only found in Heilongjiang Province [13]. IIdA14G1, IIdA17G1, IIdA18G1, and IIdA21G1 were detected only in Xinjiang [11], Beijing Municipality [24], Tibet Autonomous Region [26], and Shandong Province (data unpublished), respectively.

Neonatal dairy calf infections with different *C. parvum* subtypes can lead to diarrhea in calves in various countries and regions. Four subtypes (IIdA14G1, IIdA15G1, IIdA19G1, and IIdA20G1) found in our study can also cause diarrhea, which is consistent with previous data. Differences of virulence, varying with *Cryptosporidium* subtypes, is also no surprise as the evolution of the pathogen itself can be viewed as an emergent process [27]. In relatively separate geographic systems, such as Xinjiang, microbial pathogenesis over time can become increasingly unpredictable as a consequence of the host, microbes, and their interaction [28]. Data indicate that the *C. parvum* subtypes in dairy cattle in Xinjiang exhibit high genetic diversity and are more heterogeneous than in other research areas in China. We speculate that more subtypes may be detected in Xinjiang; therefore, more systematic epidemiological studies focusing on other animal species are needed to further clarify the genetic diversity and zoonotic transmission risk of *Cryptosporidium* spp. in this geographic region.

## 4. Materials and Methods

### 4.1. Ethics Approval

The experimental protocol was reviewed and approved by the Institutional Animal Care and Use Committee of Henan Agricultural University (approval no. LVRIAEC 2017-019) and Tarim University (approval no. ECTU 2017-0013). All farm managers provided appropriate permission prior to fecal sample collection, and no specific permits were required for the described field studies. All field studies complied with local legislative guidelines for animal care and use, and no neonatal dairy calves were injured during the fecal sample collection.

### 4.2. Study Area and Sample Collection

**Figure 1:**
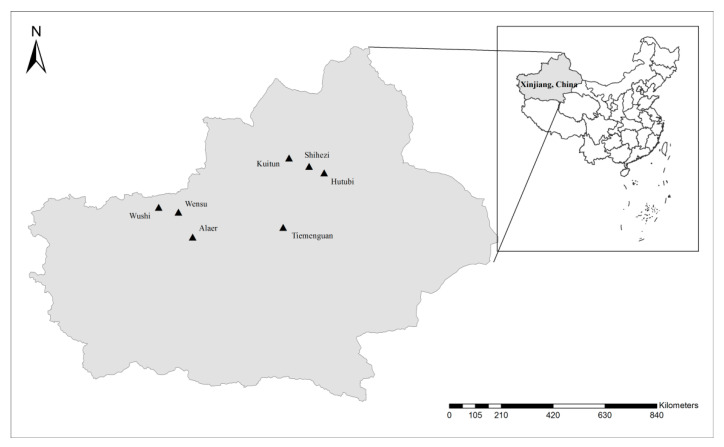
Distribution of sampling locations in Xinjiang, China. Filled triangles indicate sampling sites. No copyright permission was required. The figure was designed with ArcGIS 10.2 software. The map has been modified and assembled according to permission and attribution guidelines of the National Geomatics Center of China (http://www.ngcc.cn).

From April 2017 to April 2018, 232 fresh fecal samples (weighing approximately 20–40 g each) were randomly collected from 12 dairy cattle farms from the cities of Wushi, Alaer, Wensu, Shihezi, Hutubi, Tiemenguan, and Kuitun in Xinjiang, northwest China (Figure 1). The farms were of similar scale, consisting of 1400–1700 dairy cattle. The collected samples accounted for 50% of the number of neonatal dairy calves per farm.

All calves were 7–28 days old and separately housed in calf hutches or raised in neonatal dairy calf cowsheds. All fecal samples were collected directly from the rectum of each calf with a sterile disposable latex glove. Diarrheal status was observed according to clinical symptoms, and neonatal dairy calves were divided into two groups: those with diarrhea and those without diarrhea. The samples were transported to the laboratory and stored at 4 °C for use in subsequent molecular analyses.

### 4.3. DNA Extraction and PCR Amplification

Genomic DNA was extracted from approximately 200 mg per sample using the E.Z.N.A^®^ Stool DNA Kit (Omega Biotek Inc., Norcross, GA, USA). The DNA was stored at −20 °C prior to use in PCR amplification.

The small subunit rRNA (*SSU* rRNA) gene was used to identify the *Cryptosporidium* species using previously described primers and conditions [29]. All *SSU* rRNA PCR products were further analyzed via RFLP method using the *Ssp*I and *Mbo*II restriction enzymes (TaKaRa Shuzo Co. Ltd., Otsu, Japan) [30]. *C. parvum*-positive samples, confirmed by DNA sequencing of the PCR products, were subtyped based on the sequence analysis of the 60-kDa glycoprotein gene (*gp60*) [31]. To improve the specificity and sensitivity of the PCR assays, two replicates were used per sample, and a positive sample in two replicates was considered infected. Positive (chicken-derived *C. bailey* DNA) and negative (containing no template DNA) controls were set during the amplification. The secondary PCR products were examined via electrophoresis using 1.5% agarose gel and staining with GelRed (Biotium Inc., Hayward, CA, USA).

### 4.4. Sequencing and Data Analysis

All positive PCR products based on *SSU* rRNA and *gp60* genes were bidirectionally sequenced on an ABI PRISM™ 3730 XL DNA Analyzer using the BigDye Terminator v3.1 Cycle Sequencing Kit (Applied Biosystems, Foster City, CA, USA). Sequence accuracy was confirmed via two-directional sequencing, and by sequencing additional PCR products when necessary. The raw sequences obtained were edited with DNAstar Lasergene Editseq version 7.1.0 (http://www.dnastar.com/) and aligned with reference sequences downloaded from GenBank (http://www.ncbi.nlm.nih.gov/) using Clustal X version 2.1 (http://www.clustal.org/).

### 4.5. Nucleotide Sequence Accession Numbers

Representative *C. parvum* subtype nucleotide sequences identified in the neonatal dairy calves were submitted to GenBank at the National Center for Biotechnology Information under accession numbers MT680896–MT680900.

### 4.6. Statistical Analysis

SPSS ver. 22.0 (IBM Corp., Armonk, NY, USA) was used for all statistical analyses. *Cryptosporidium* infection rates were calculated and compared using the chi-square test. Differences were considered significant at *p* < 0.05.

## 5. Conclusions

Four *C. parvum* IId subtypes (IIdA14G1, IIdA15G1, IIdA19G1, and IIdA20G1) were detected, and the data indicate that *C. parvum* subtypes from neonatal dairy calves in Xinjiang have high genetic diversity. The statistically significant differences in *Cryptosporidium* prevalence between neonatal dairy calves with and without diarrhea indicate that *C. parvum* may be a major contributor to diarrheal disease in neonatal dairy calves. Our findings suggest that neonatal dairy calves may be a potential source of human *Cryptosporidium* infection and further evidence the uniqueness of *C. parvum* IId subtypes in dairy cattle in China.

## Figures and Tables

**Table 1:** Occurrence of *Cryptosporidium* species and subtypes in neonatal dairy calves in Xinjiang, China.

Farm	Number of Samples	Number of Positive Samples (%)	*Cryptosporidium* Species and Subtypes (no.)
Wushi1	11	7 (63.6)	*C. parvum* (7), IIdA20G1 (7)
Wushi2	24	8 (33.3)	*C. parvum* (8) ^1^, IIdA20G1 (7)
Alaer	19	7 (36.8)	*C. parvum* (7), IIdA14G1 (6), IIdA15G1 (1)
Wensu1	17	7 (41.2)	*C. parvum* (7), IIdA15G1 (7)
Wensu2	16	6 (37.5)	*C. parvum* (6), IIdA15G1 (6)
Shihezi1	23	6 (26.1)	*C. parvum* (6), IIdA14G1 (1), IIdA15G1 (5)
Shihezi2	21	8 (38.1)	*C. parvum* (4), IIdA14G1 (3), IIdA15G1 (1); *C. parvum + C. ryanae* (4) ^3^, IIdA14G1 (3), IIdA15G1 (1)
Shihezi3	25	10 (40.0)	*C. parvum* (6), IIdA14G1 (1), IIdA15G1 (5); *C. parvum + C. ryanae* (4) ^3^, IIdA14G1 (3), IIdA15G1 (1)
Hutubi1	21	8 (38.1)	*C. parvum* (8), IIdA19G1 (8)
Hutubi2	20	5 (25.0)	*C. parvum* (5), IIdA19G1 (5)
Tiemenguan	15	10 (66.7)	*C. parvum* (9) ^1^, IIdA15G1 (8); *C. bovis + C. ryanae* (1) ^3^
Kuitun	20	7 (35.0)	*C. parvum* (7), IIdA20G1 (7)
Total	232	89 (38.4)	*C. parvum* (80) ^2^, IIdA14G1 (11), IIdA15G1 (33), IIdA19G1 (13), IIdA20G1 (21); *C. parvum + C. ryanae* (8) ^3^, IIdA14G1 (6), IIdA15G1 (2); *C. bovis + C. ryanae* (1) ^3^

^1^ One isolate unsuccessfully subtyped. ^2^ Two isolates unsuccessfully subtyped. ^3^ Mixed infections.

**Table 2:** *Cryptosporidium* species and subtypes according to clinical symptoms in neonatal dairy calves in the present study.

Clinical Symptom	Farm	Number of Positive/Number of Examined (%)	*Cryptosporidium* Species and Subtypes (no.)
Diarrhea	Wushi1	6/8 (75.0)	*C. parvum* (6), IIdA20G1 (6)
	Wushi2	5/11 (45.5)	*C. parvum* (5) ^1^, IIdA20G1 (4)
	Alaer	3/7 (42.9)	*C. parvum* (3), IIdA14G1 (3)
	Wensu1	4/11 (36.4)	*C. parvum* (4), IIdA15G1 (4)
	Wensu2	6/11 (54.5)	*C. parvum* (6), IIdA15G1 (6)
	Shihezi1	5/9 (55.6)	*C. parvum* (5), IIdA15G1 (5)
	Shihezi2	3/4 (75.0)	*C. parvum* (2), IIdA14G1 (2); *C. parvum+C. ryanae* (1), IIdA14G1 (1)
	Shihezi3	4/7 (57.1)	*C. parvum* (4), IIdA14G1 (1), IIdA15G1 (3)
	Hutubi1	2/4 (50.0)	*C. parvum* (2), IIdA19G1 (2)
	Hutubi2	2/9 (22.2)	*C. parvum* (2), IIdA19G1 (2)
	Tiemenguan	7/9 (77.8)	*C. parvum* (7) ^1^, IIdA15G1 (6)
	Kuitun	4/7 (57.1)	*C. parvum* (4), IIdA20G1 (4)
	Subtotal 1	51/97 (52.6)	*C. parvum* (50) ^2^, IIdA14G1 (6), IIdA15G1 (24), IIdA19G1 (4), IIdA20G1 (14); *C. parvum + C. ryanae* (1), IIdA14G1 (1)
No diarrhea	Wushi1	1/3 (33.3)	*C. parvum* (1), IIdA20G1 (1)
	Wushi2	3/13 (23.1)	*C. parvum* (3), IIdA20G1 (3)
	Alaer	4/12 (33.3)	*C. parvum* (4), IIdA14G1 (3), IIdA15G1 (1)
	Wensu1	3/6 (50.0)	*C. parvum* (3), IIdA15G1 (3)
	Wensu2	0/5 (0)	
	Shihezi1	1/14 (7.1)	*C. parvum* (1), IIdA14G1 (1)
	Shihezi2	5/17 (29.4)	*C. parvum* (2), IIdA14G1 (1), IIdA15G1 (1); *C. parvum+C. ryanae* (3), IIdA14G1 (2), IIdA15G1 (1)
	Shihezi3	6/18 (33.3)	*C. parvum* (2), IIdA15G1 (2);*C. parvum+C. ryanae* (4), IIdA14G1 (3), IIdA15G1 (1)
	Hutubi1	6/17 (35.3)	*C. parvum* (6), IIdA19G1 (6)
	Hutubi2	3/11 (27.3)	*C. parvum* (3), IIdA19G1 (3)
	Tiemenguan	3/6 (50.0)	*C. parvum* (2), IIdA15G1 (2); *C. bovis+C. ryanae* (1)
	Kuitun	3/13 (23.1)	*C. parvum* (3), IIdA20G1 (3)
	Subtotal 2	38/135 (28.1)	*C. parvum* (30), IIdA14G1 (5), IIdA15G1 (9), IIdA19G1 (9), IIdA20G1 (7); *C. parvum + C. ryanae* (7), IIdA14G1 (5), IIdA15G1 (2); *C. bovis + C. ryanae* (1)

^1^ One isolate unsuccessfully subtyped. ^2^ Two isolates unsuccessfully subtyped.

**Table 3:** Geographical distribution of *C*. *parvum* IId subtype family from cattle in China.

Subtype	Breed	Number of Positive	Province (no.)	Reference
IIdA14G1	Dairy cattle	21	Xinjiang (21)	[22], this study
IIdA15G1	Dairy cattle	165	Ningxia (85) ^1^, Xinjiang (46), Heilongjiang (24), Sichuan (7), Gansu (1), Beijing (1) Shandong (1) ^2^	[7,11,13,18,19,20,24], this study
	Yak	3	Gansu (2), Qinghai (1)	[26]
IIdA17G1	Dairy cattle	1	Beijing (1)	[24]
IIdA18G1	Yak	1	Qinghai (1)	[26]
IIdA19G1	Dairy cattle	250	Jiangsu (77) ^1^, Henan (67), Shanghai (66), Xinjiang (13), Guangdong (10), Hebei (5), Tianjin (5), Shandong (5) ^2^, Beijing (1), Heilongjiang (1)	[10,12,16,22,23,24], this study
	Yak	1	Tibet (1)	[26]
IIdA20G1	Dairy cattle	69	Heilongjiang (48), Xinjiang (21)	[13], this study
IIdA21G1	Dairy cattle	4	Shandong (4) ^2^	

^1^ On farm of cryptosporidiosis outbreak. ^2^ Data unpublished.
